# Performance and safety of the induced sputum procedure in young children in Malawi: a prospective study

**DOI:** 10.1093/trstmh/trab151

**Published:** 2021-09-29

**Authors:** Wongani Nyangulu, Herbert Thole, Angella Chikhoza, Mike Msakwiza, James Nyirenda, Mphatso Chisala, Pui-Ying Iroh Tam

**Affiliations:** Paediatrics and Child Health Research Group, Malawi-Liverpool Wellcome Trust, Queen Elizabeth Central Hospital, College of Medicine, P.O. Box 30096, Chichiri, Blantyre 3, Malawi; Paediatrics and Child Health Research Group, Malawi-Liverpool Wellcome Trust, Queen Elizabeth Central Hospital, College of Medicine, P.O. Box 30096, Chichiri, Blantyre 3, Malawi; Paediatrics and Child Health Research Group, Malawi-Liverpool Wellcome Trust, Queen Elizabeth Central Hospital, College of Medicine, P.O. Box 30096, Chichiri, Blantyre 3, Malawi; Paediatrics and Child Health Research Group, Malawi-Liverpool Wellcome Trust, Queen Elizabeth Central Hospital, College of Medicine, P.O. Box 30096, Chichiri, Blantyre 3, Malawi; Paediatrics and Child Health Research Group, Malawi-Liverpool Wellcome Trust, Queen Elizabeth Central Hospital, College of Medicine, P.O. Box 30096, Chichiri, Blantyre 3, Malawi; Paediatrics and Child Health Research Group, Malawi-Liverpool Wellcome Trust, Queen Elizabeth Central Hospital, College of Medicine, P.O. Box 30096, Chichiri, Blantyre 3, Malawi; Paediatrics and Child Health Research Group, Malawi-Liverpool Wellcome Trust, Queen Elizabeth Central Hospital, College of Medicine, P.O. Box 30096, Chichiri, Blantyre 3, Malawi; Department of Paediatrics, University of Malawi, College of Medicine, Private Bag 360, Chichiri, Blantyre 3, Malawi; Department of Clinical Sciences, Liverpool School of Tropical Medicine, L3 5QA, Liverpool, UK

**Keywords:** children, correlation of data, cryptosporidiosis, diagnosis, Malawi, sputum

## Abstract

**Background:**

Collecting sputum specimens is a challenge in infants and young children. We assessed the performance and safety of induced sputum (IS) collection in this population, embedded in a prospective study evaluating respiratory cryptosporidiosis in Malawian children with diarrheal disease.

**Methods:**

We assessed the sputum quality and correlation with detection of *Cryptosporidium* spp. and evaluated safety and adverse events in 162 children.

**Results:**

Among 159 stool specimens tested, 34 (21%, 95% CI 15.0 to 28%) were positive for *Cryptosporidium* spp. There were 160 IS and 161 nasopharyngeal (NP) specimens collected. IS and NP specimen collection was performed for each patient. The majority of IS specimens (122/147; 83%) were clear in appearance and 132/147 (90%) were of good quality. Among the respiratory specimens tested, 10 (6.3%, 95% CI 2.5 to 10%) IS and 4 (3%, 95% CI 0 to 5%) NP were positive for *Cryptosporidium* spp. When stool cryptosporidium PCR was the gold standard, IS PCR sensitivity was higher (29%, 95% CI 22 to 37%) compared with NP PCR (12%, 95% CI 7 to 17%) for detection of *Cryptosporidium* spp. One (0.4%) adverse event occurred, consisting of a drop in oxygen saturations at the 30-min postprocedure evaluation. Consciousness level, median respiratory rate and oxygen saturations were unchanged, before or after IS.

**Conclusions:**

IS provides good quality specimens, is more sensitive than NP specimens for diagnosis of respiratory cryptosporidiosis, and collection can be performed safely in children hospitalized with diarrheal disease.

## Introduction

Sputum specimens are routinely collected for the diagnosis of respiratory diseases in adults.^1^ However, this is more challenging in infants and young children^1^ who require sputum induction and/or gastric aspiration to obtain diagnostic specimens.^2^ Children have difficulty in producing an adequate specimen, difficulty with expectoration, a tendency to swallow sputum and have a high nasopharyngeal (NP) carriage of potential contaminants, including *Streptococcus pneumoniae* (19–38%), *Haemophilus influenzae* (13–25%), *Moraxella catarrhalis* (22–39%) and *Staphylococcus aureus* (16–36%).^1,3,4^

In Malawi, gastric aspiration is the standard method for obtaining pediatric sputum specimens in public hospitals. These facilities are usually under-resourced, lack equipment for induced sputum (IS) collection and staff trained to conduct IS. IS procedures are mostly performed in research settings. However, IS has a better diagnostic yield for pulmonary TB compared with gastric aspiration.^5,6^ It is also safe and well tolerated with minimal adverse events after the procedure, even in severely ill children.^7,8^ Few studies evaluating the safety of IS have been performed in low-resource settings,^8,9^ and none have been conducted in a public tertiary hospital in Malawi.

In this study, we assess the performance and safety of IS collection in infants and young children enrolled in a prospective study evaluating respiratory cryptosporidiosis in children hospitalized with diarrheal disease.^10^

## Materials and Methods

### Participants

The participants were children aged 2–24 mo hospitalized with a primary diagnosis of diarrhea as part of a study evaluating the role of respiratory cryptosporidiosis in pediatric diarrheal disease.^10,11^ All hospitalized children with a primary diagnosis of diarrhea were recruited consecutively into the study. The inclusion criteria were: (1) male or female; (2) age 2–24 mo; (3) hospitalized with a primary diagnosis of diarrhea (defined as ≥3 loose stools within 24 h); (4) living within 15 km radius of the Blantyre district; and (5) a parent or guardian providing consent for enrollment. Children with dysentery (defined as visible blood in loose stools) were excluded. All enrolled patients had IS, NP and stool specimens collected for *Cryptosporidium* spp. testing at enrollment, and then every 2 wk up to 8 wk postenrollment if any of the enrollment specimens were positive.

### Specimen collection

IS was only performed in patients without any contraindications as described in the published protocol. Contraindications for induced sputum included: severe hypoxia <92% on supplemental oxygen; inability to protect airways; severe bronchospasm upon admission (defined as severe hypoxia <92% after appropriate bronchodilator therapy, with other markers of respiratory distress); seizure within the past 24 h; or deemed inappropriate by the clinician for another reason (e.g. midface trauma, inhalational injury, congestive heart failure).^10^

IS was performed as follows: patients were given nebulized salbutamol followed by 3% sodium chloride mist inhaled for 5–15 min. A sterile catheter connected to a suction machine was passed through the nose into the posterior nasopharynx. Suction was applied only once to aspirate contents and immediately stopped. The catheter was withdrawn and flushed using sterile normal saline. IS was stopped if the child could not tolerate the procedure or if there was an adverse reaction. The child was followed up at 30 min, 2 h and 4 h postprocedure to monitor for any adverse events.

### Laboratory procedures

Sputum quality was assessed at enrollment by microscopy, noting the appearance (clear vs cloudy) and counting the number of squamous epithelial cells (SECs) per low power field (LPF). Good quality sputum was classified as ≤10 SECs per LPF. The number of neutrophils per LPF was also recorded. PCR testing was performed to detect *Cryptosporidium* spp. using methods described elsewhere.^10^

### Statistical analysis

Data were analyzed using STATA 13 (Stata Corp, USA). We reported proportions for all categorical variables, and median and IQR for non-normally distributed continuous variables. Performance was measured by calculating the sensitivity and specificity of PCR using stool and sputum cryptosporidium PCR, respectively, as the gold standards. Results are reported at the 5% significance level.

## Results

A total of 162 children were enrolled in the study. Of these, 28 had >1 IS performed (159 had one IS performed, one child had two IS performed, one child had three IS performed, four children had four IS performed and 22 children had five IS performed), and a total of 262 IS procedures were carried out. The median age of enrolled children was 11 (IQR 8–14) mo and 95 (59%) were male. Table 1 shows the health status of children before the IS procedure. Most of the children (64%) looked either unwell or uncomfortable, or sick and requiring hospitalization.

**Table 1.** Health status of children before and after the induced sputum procedure

Characteristic	Immediately before	Immediately after
Fever (temperature >37.5ºC; %)	18 (11)	ND
Oxygen saturations (%), median (IQR)	99 (98–100)	98 (98–99)
Respiratory rate (bpm), median (IQR)	35 (32–38)	36 (34–38)
Looking sick, requiring referral to the hospital (%)	52 (32)	ND
Looking unwell, uncomfortable (%)	52 (32)	ND
Looking comfortable, well and playful (%)	58 (36)	ND
Receiving supplemental oxygen via nasal prongs (%)	3 (2)	3 (2)
Receiving intravenous fluid (%)	38 (24)	ND
Bipedal edema	5 (3)	ND
Consciousness level (AVPU* score)		
1. No. (%) V, P or U	0 (0)	0 (0)
2. No. (%) with any decrease from baseline	ND	0 (0)

Abbreviation: ND, not done.

* A=alert, V=verbal, P=pain, U=unresponsive.

There were 147 induced sputum specimens with documented appearance and cell counts. The majority of sputum specimens (122/147; 83%) were clear, and only a few were either cloudy or mucoid (16% and 1%, respectively). Most of the specimens (132/147; 90%) were of good quality with <10 SECs per LPF. Only one specimen had >10 neutrophils per LPF. None of these characteristics was significantly associated with *Cryptosporidium* spp. positivity (Supplementary material).

Among 159 stool specimens tested at enrollment, 34 (21%, 95% CI 15 to 28%) were positive for *Cryptosporidium* spp. Among 160 IS and 161 NP specimens that were tested, 10 (6%, 95% CI 3 to 10%) and 4 (3%, 95% CI 0 to 5%), respectively, were positive. When stool cryptosporidium PCR was used as the gold standard, the sensitivity of IS PCR compared with NP PCR for detection of *Cryptosporidium* spp. was 29% (95% CI 22 to 37%) compared with 12% (95% CI 7 to 17%). Both tests had a specificity of 100% (95% CI 100 to 100%). When sputum PCR was used as the gold standard, NP PCR had a sensitivity of 40% (95% CI 32 to 48%).

There was one adverse event following all IS procedures representing a risk of 0.4%: a child experienced a drop in oxygen saturations below 92% at the 30-min evaluation post-IS. The child did not present with any respiratory symptoms at hospitalization. The patient was managed supportively and oxygen saturations at subsequent follow-up intervals were normal without any interventions.

Median oxygen saturations and respiratory rates remained stable before and after IS. There was no change in the number of children requiring supplemental oxygen immediately before IS to 4 h after the procedure. There were 24 children (15%) who had tachypnea immediately before IS. Immediately after IS, 24 children (15%) had tachypnea. At 30 min, 2 h and 4 h postprocedure, 14 (9%), 11 (7%) and 14 (9%) children had tachypnea, respectively. A table showing the proportion of tachypnea at the different time points by age group is provided (Supplementary Table 10). All episodes resolved spontaneously. No children experienced a decreased level of consciousness before or after IS. There were three children who were on oxygen therapy before the procedure. However, their oxygen saturations were >92% before and after the procedure.

## Discussion

This is one of a few studies comparing the performance of IS with NP for the detection of respiratory parasites, and one of the first performed in a low-resource setting.

Other studies of IS have established the procedure to provide good quality sputum specimens with a high microbial yield in children.^4^ In a prospective study in South Africa, IS was performed in 142 children. Compared with gastric lavage, the yield for *Mycobacterium tuberculosis* was 4% higher for IS specimens (95% CI 0 to 5.6%; p=0.08).^12^ In another study in The Netherlands of children admitted with acute lower respiratory infection, 98 children had IS performed. A total (89/98; 91%) had good quality sputum, and bacterial pathogens were isolated from 22/89 (25%).^13^ In India, IS was performed in 120 children diagnosed with pneumonia. There was good quality sputum in 64 children (53.3%). Among these, 45/64 (70%) had isolates of *Klebsiella pneumoniae* (38.2%) and *Str. pneumoniae* (14.8%) or other bacteria.^9^

The IS procedure in young children hospitalized with diarrheal disease was safe and well tolerated in our study in both well and unwell subjects. Adverse events were rare and involved only one subject (0.4%), and this is consistent with studies conducted elsewhere, including the PERCH study, where the proportion of adverse events was 0.34%,^8^ even although that study recruited children with moderate to severe pneumonia. The PERCH study team recommended safety monitoring during and after IS up to 2 h postprocedure, with close attention paid to oxygen saturation in severely ill children with pneumonia.^8^ PERCH had a larger sample size and recorded more adverse events than our study. Therefore, our findings do not support the need for postprocedure follow-up, although it is important to note that we did not recruit and conduct IS on children with a primary respiratory diagnosis.

We demonstrated that well-trained staff in public hospitals can conduct IS with good sputum quality for the diagnosis of respiratory pathogens. We also demonstrated that this procedure is safe in a low-resource setting. This has implications for practice in those hospitals where IS is not regularly performed due to a lack of trained staff and materials, including hypertonic saline. Medical and nursing staff can be trained to conduct IS safely with good diagnostic yield and the materials required for the procedure are relatively inexpensive. The low rate of adverse events among patients with a primary diarrheal diagnosis suggests that clinical protocols with close monitoring of patients after the procedure may not be necessary.

The procedure was also acceptable to parents and guardians of children admitted in hospital. In our study, none of the parents or guardians were familiar with the IS procedure. All were worried about the procedure, thought it was uncomfortable and would cause pain. However, after detailed explanation of the procedure, all the parents and guardians provided consent. Those who had repeat procedures performed did not express similar misgivings towards IS during the follow-up study visits.

The main limitation of the current study is a smaller than anticipated sample size. Based on the expected sample size for the main study and the expected *Cryptosporidium* spp. positivity rate, we expected to conduct >300 IS procedures.^10^ The emergence of the severe acute respiratory syndrome-coronavirus 2 global pandemic resulted in suspension of all study activities.

## Conclusions

In summary, we demonstrated that the IS procedure in young children hospitalized with diarrheal disease has a higher sensitivity compared with NP swabs for diagnosis of respiratory cryptosporidiosis, and is safe and acceptable in a low-resource setting. Our study and these findings suggest that IS can perform well and be performed safely in our setting for the diagnosis of respiratory pathogens.

## Supplementary Material

trab151_Supplemental_File

## Data Availability

Data will be made available upon reasonable request to the primary study principal investigator (P-YIT).

## References

[1] Murdoch DR, Morpeth SC, Hammitt LL et al. Microscopic analysis and quality assessment of induced sputum from children with pneumonia in the PERCH Study. Clin Infect Dis. 2017;64(suppl_3):S271–9. 28575360 10.1093/cid/cix083 PMC5447851

[2] Zar HJ, Hanslo D, Apolles P et al. Induced sputum versus gastric lavage for microbiological confirmation of pulmonary tuberculosis in infants and young children: a prospective study. Lancet. 2005;365(9454):130–4. 15639294 10.1016/S0140-6736(05)17702-2

[3] Grant LR, Hammitt LL, Murdoch DR et al. Procedures for collection of induced sputum specimens from children. Clin Infect Dis. 2012;54(suppl_2):S140–5. 22403228 10.1093/cid/cir1069 PMC3297553

[4] Lahti E, Peltola V, Waris M et al. Induced sputum in the diagnosis of childhood community-acquired pneumonia. Thorax. 2009;64(3):252–7. 19052043 10.1136/thx.2008.099051

[5] Heym B, Beauchet A, Ngo MT et al. Sputum induction versus gastric washing for the diagnosis of pulmonary mycobacterial disease. Eur Respir J. 2010;36(2):448–50. 20675783 10.1183/09031936.00192209

[6] Brown M, Varia H, Bassett P et al. Prospective study of sputum induction, gastric washing, and bronchoalveolar lavage for the diagnosis of pulmonary tuberculosis in patients who are unable to expectorate. Clin Infect Dis. 2007;44(11):1415–20. 17479935 10.1086/516782

[7] Planting NS, Visser GL, Nicol MP et al. Safety and efficacy of induced sputum in young children hospitalised with suspected pulmonary tuberculosis. Int J Tuberc Lung Dis. 2014;18(1):8–12. 24365546 10.5588/ijtld.13.0132

[8] DeLuca AN, Hammitt LL, Kim J et al. Safety of induced sputum collection in children hospitalized with severe or very severe pneumonia. Clin Infect Dis. 2017;64(suppl_3):S301–8. 28575356 10.1093/cid/cix078 PMC5447836

[9] Kurade A, Dhanawade S, Shetti S. Induced sputum as a diagnostic tool in pneumonia in under five children—a hospital-based study. J Trop Pediatr. 2018;64(6):510–5. 29415185 10.1093/tropej/fmx106

[10] Nyangulu W, Van Voorhis W, Iroh Tam P-Y. Evaluating respiratory cryptosporidiosis in pediatric diarrheal disease: protocol for a prospective, observational study in Malawi. BMC Infect Dis. 2019;19(1):728. 31426759 10.1186/s12879-019-4380-x PMC6701119

[11] Iroh Tam P-Y, Chisala M, Nyangulu W et al. Respiratory cryptosporidiosis in Malawian children with diarrheal disease. PLoS Negl Trop Dis. 2021;15(7):e0009643. 34329296 10.1371/journal.pntd.0009643 PMC8357119

[12] Zar HJ, Tannenbaum E, Apolles P et al. Sputum induction for the diagnosis of pulmonary tuberculosis in infants and young children in an urban setting in South Africa. Arch Dis Child. 2000;82(4):305–8. 10735837 10.1136/adc.82.4.305 PMC1718283

[13] Bart IY, Mourits M, van Gent R et al. Sputum induction in children is feasible and useful in a bustling general hospital practice. Glob Pediatr Heal. 2016;3:2333794×16636504. 10.1177/2333794X16636504 PMC4905149 27336008

